# Biological Surface Coating and Molting Inhibition as Mechanisms of TiO_2_ Nanoparticle Toxicity in *Daphnia magna*

**DOI:** 10.1371/journal.pone.0020112

**Published:** 2011-05-27

**Authors:** André Dabrunz, Lars Duester, Carsten Prasse, Frank Seitz, Ricki Rosenfeldt, Carsten Schilde, Gabriele E. Schaumann, Ralf Schulz

**Affiliations:** 1 Institute for Environmental Sciences, University of Koblenz-Landau, Landau, Germany; 2 Department of Aquatic Chemistry, Federal Institute of Hydrology, Koblenz, Germany; 3 Institute for Particle Technology, Technical University of Braunschweig, Braunschweig, Germany; George Mason University, United States of America

## Abstract

The production and use of nanoparticles (NP) has steadily increased within the last decade; however, knowledge about risks of NP to human health and ecosystems is still scarce. Common knowledge concerning NP effects on freshwater organisms is largely limited to standard short-term (≤48 h) toxicity tests, which lack both NP fate characterization and an understanding of the mechanisms underlying toxicity. Employing slightly longer exposure times (72 to 96 h), we found that suspensions of nanosized (∼100 nm initial mean diameter) titanium dioxide (nTiO_2_) led to toxicity in *Daphnia magna* at nominal concentrations of 3.8 (72-h EC_50_) and 0.73 mg/L (96-h EC_50_). However, nTiO_2_ disappeared quickly from the ISO-medium water phase, resulting in toxicity levels as low as 0.24 mg/L (96-h EC_50_) based on measured concentrations. Moreover, we showed that nTiO_2_ (∼100 nm) is significantly more toxic than non-nanosized TiO_2_ (∼200 nm) prepared from the same stock suspension. Most importantly, we hypothesized a mechanistic chain of events for nTiO_2_ toxicity in *D. magna* that involves the coating of the organism surface with nTiO_2_ combined with a molting disruption. Neonate *D. magna* (≤6 h) exposed to 2 mg/L nTiO_2_ exhibited a “biological surface coating” that disappeared within 36 h, during which the first molting was successfully managed by 100% of the exposed organisms. Continued exposure up to 96 h led to a renewed formation of the surface coating and significantly reduced the molting rate to 10%, resulting in 90% mortality. Because coating of aquatic organisms by manmade NP might be ubiquitous in nature, this form of physical NP toxicity might result in widespread negative impacts on environmental health.

## Introduction

The steady increase in the production and use of synthetic nanoparticles (NP) in consumer products [1], such as cosmetics or paint, is likely to result in an unintentional release of NP into the environment, causing unknown risks to human and wildlife health [2], [3]. Nanosized titanium dioxide (nTiO_2_) is one of the most important inorganic NP with regards to production volume and multitude of uses [4], [5]. The majority of NP toxicity studies so far have been conducted with nTiO_2_, its agglomerates, and the water flea *Daphnia magna* Straus, an important standard test organism in aquatic ecotoxicity testing [6]. Most of these studies found acute effect levels to be >100 mg/L [7]. In contrast to this, Lovern and Klaper [8] reported a 48-h LC_50_ of 5.5 mg/L; however, the NP product used and particle size distributions over the course of the experiment were not provided in the publication. Unfortunately, available nTiO_2_ toxicity studies often lack information on NP characteristics and data regarding the verification of the results, e.g., particle size distribution and concentration in the test media. The only study using nTiO_2_ and *D. magna* that provided both particle size distribution and concentration measurements of nTiO_2_ found a 72-h EC_50_ of 1.62 mg/L [9]. However, the control effects in this study exceeded the maximum level of the OECD acute toxicity testing guideline 202 of 10% [10].

The recent application of probabilistic material flow analysis of engineered nanomaterials predicted maximum surface water concentrations of 0.085 µg/L nTiO_2_, which are well below those implicated in environmental risks [11]. Following another study measuring Ti in centrifuged samples taken from urban runoff, including exterior facades wash-off [12], a calculated concentration of about 0.073 µg/L nTiO_2_ (≤100 nm fraction) is present in the urban runoff entering a creek.

Thus far, an underlying principle in environmental risk assessment has been that the toxicity of chemicals is mainly driven by the exposure dose [13] due to a focus on test protocols originally developed for traditional chemicals, such as pesticides. However, for nanomaterials, particle size and particle surface characteristics appear to be at least as important as the available dose in driving the extent of potential toxic effects [14]. The persisting lack of understanding of NP toxicity in aquatic organisms is, to a large extent, a consequence of huge gaps in knowledge regarding the underlying mechanisms. Due to the photocatalytic properties of nTiO_2_, the formation of reactive oxygen species has been suggested as a mode of toxic action [2], although studies focusing on the photocatalytic aspects of nTiO_2_ toxicity did not result in clear concentration effect relations for *D. magna* [15]. Therefore, a mechanistic approach for understanding the chemistry and mechanisms underlying the biological effects of NP is needed [16].

The present study employed *D. magna* as a representative of zooplankton, a key group for the food web structure and the matter turnover in lake ecosystems [17]. In contrast to most previous acute toxicity tests using nTiO_2_ and *D. magna*, a 96-h exposure was used instead of the standard 48-h test duration, which hardly ever showed effects on *D. magna* [6]. Such a 96-h toxicity test setup with *D. magna* using mortality and growth as endpoints has previously been suggested for general chemical testing [18]. Moreover, a recent study showed an increased toxicity of nTiO_2_ following an exposure of 72 h [9], illustrating the potential of a prolonged acute test duration.

Using a variety of experiments, we assessed the effects of different TiO_2_ size classes (Exp. 1) and concentrations during 96-h acute toxicity testing (Exp. 2) on *D. magna*. The present study also aimed to characterize the dissipation of nTiO_2_ from the water phase in order to more precisely define the exposure via the water phase throughout the test duration (Exp. 3). Additionally, the NP suspensions were characterized in the course of each experiment. Finally, we evaluated the mechanisms of toxicity by a qualitative description of NP - *Daphnia* surface interactions and by quantifying the effects of nTiO_2_ on the molting of *D. magna* (Exp. 4).

## Methods

### Suspension and particle size characterization

Uncoated titanium dioxide nanoparticles (A-100, Crenox GmbH, Krefeld, Germany) were purchased as a powder in pure anatase form. The product had an advertised primary particle size of 6 nm, and the surface area (Brunner–Emmett–Teller; BET) was approximately 230 m^2^/g. From this product additive free, size homogenized, stable suspensions were obtained by stirred media milling (PM2, Bühler AG, Switzerland) followed by centrifugation to remove residual coarse material. Size distributions in undiluted, monodisperse stock suspensions showing average diameters of ∼100 nm and ∼200 nm were determined by dynamic light scattering (Delsa™ Nano C, Beckman Coulter, Krefeld) prior to each experiment (Table 1). Suspensions used were shown to be stable for over a month with a size variation of the average diameter below ±5% [19].

**Table 1:** Particle characterization of TiO_2_ suspensions used in the experiments of the present study.

Number	Name of Experiment	Mean diameter	D_10_ 1	D_90_ 1	PI^2^
Exp. 1	Size effects	101 nm	60 nm	188 nm	0.150
Exp. 1	Size effects	205 nm	107 nm	444 nm	0.234
Exp. 2	Dose effects	94 nm	59 nm	162 nm	0.126
Exp. 3	NP dissipation	94 nm	59 nm	162 nm	0.126
Exp. 4	Molting inhibition	96 nm	59 nm	167 nm	0.124

1 Values indicate 10% and 90% particle fractions below the respective size.

2 Polydispersity index.

#### Test organism

*D. magna* was obtained from Eurofins-GAB laboratories (Niefern-Oeschelbronn, Germany) and kept in permanent culture within climate controlled chambers at 20±1°C on a 16∶8 h (light:dark) photoperiod. Animals were cultured in M4 medium [10] that was renewed three times a week. Animals were fed with green algae *Desmodesmus* sp. on a daily basis.

### Acute toxicity tests (Exp. 1 and 2)

The acute tests focusing on either size- or concentration-dependent toxicity of nTiO_2_ were conducted based on OECD guideline 202 with prolonged observation intervals of 72 h and 96 h. To reduce variability and ensure survival rates >90% in the control treatments even after 96 hours without feeding, neonates were fed algae with an equivalent of about 6 µg C per individual for 90 min prior to the test. Five neonate daphnids (age ≤24 h) were exposed to nTiO_2_ in 150 mL glass beakers filled with 50 mL of ISO test medium (water column height: 2.7 cm). Each treatment group consisted of six replicates. Individuals that did not move following agitation of the test beakers were considered immobile, and the number of immobile individuals was recorded after 24 h and 48 h (standard design) and after 72 h and 96 h (prolonged observation intervals).

During Exp. 1, which investigated TiO_2_ size-related toxicities, we investigated suspensions of ∼200 nm and ∼100 nm in mean diameter (Table 1) at a concentration of 2 mg/L. The examinations of concentration- and exposure time-related toxicities (Exp. 2) were performed by exposing *D. magna* to nTiO_2_ (∼100 nm) at 0.5, 1, 2, 4 and 8 mg/L plus control for up to 96 h.

### nTiO_2_ dissipation from medium (Exp. 3) and photographic monitoring

To examine the dissipation of nTiO_2_ from the aqueous phase, triplicate samples from each concentration (0.5, 1, 2, 4, and 8 mg/L nTiO_2_) were taken from the centre of the water column. During sampling, special attention was paid to avoid particle resuspension. Water samples obtained after 0, 1, 2, 3, 6, 12, 24, 48, 72 and 96 h were analyzed for the Ti concentration (mass 47) using a Quadropole ICP-MS (XSeries2, Thermo Fischer Scientific, Dreieich) equipped with a FAST autosampler (ESI, Thermo Fischer Scientific, Dreieich), a peek spray chamber (Thermo Fischer Scientific, Dreieich) and a robust Mira Mist peek nebuliser (Burgener, Berkshire). The instrument was run in the collision cell mode with 5 ml He/H_2_ cell gas in order to avoid polyatomic interferences (e.g., PO^+^ or SiOH^+^). All results obtained were well above the limits of detection (0.05–0.38 µg Ti/L). Pictures showing biological surface coating were obtained using an A1 Axio Scope microscope (Carl Zeiss GmbH, Oberkochen) at a 50-fold magnification with an impinging light setup. SEM-EDX pictures were taken using a Quanta 250 electron microscope (FEI Company, Eindhoven) at a 1200-fold magnification.

### Daphnia molting (Exp. 4)

Within the first 96 h, a juvenile *D. magna* kept at 20±1°C regularly molts once when starved [20] and up to 3 times under optimal feeding conditions (unpublished own observations). The influence of nTiO_2_ (2 mg/L; ∼100 nm) on the molting of *D. magna* was assessed by exposing more precisely age-synchronized neonates (age ≤6 h) to conditions similar to Exp. 1 and 2. Each beaker (n = 20) contained only one specimen in order to assess molting at the individual level. The occurrence of exuvia (outer shell) and immobilization was recorded every 6 h for a total of 96 h.

### Statistical analysis

Statistical analyses were carried out using the statistical software package R (version 2.10.1.). EC_50_ values were calculated using the drc package [21]. Because the data from the size and molting experiments were not normally distributed, two sided Wilcoxon rank tests were performed, and a difference was considered to be statistically significant at p-values <0.05.

## Results

### Particle size dependency of TiO_2_ toxicity

**Figure 1:**
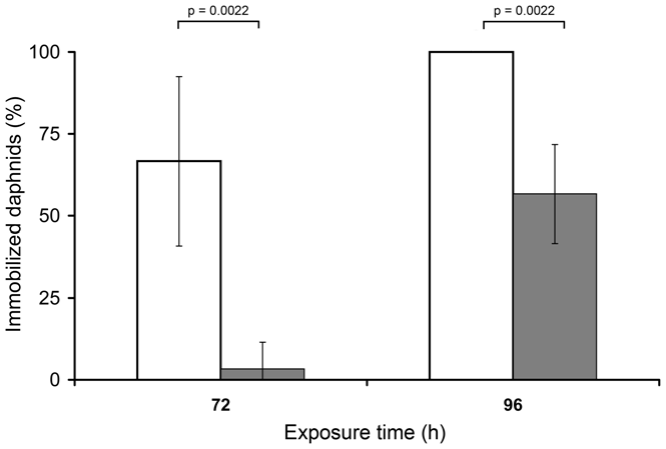
Mean immobilization (±SD; n = 6) of *D. magna* exposed to 2 mg/L TiO_2_ of different sizes. White bars: ∼200 nm TiO_2_; grey bars: ∼100 nm nTiO_2_.

During Exp. 1, immobilization of animals was never observed within the standard intervals of 24 h and 48 h. However, after 72 h, immobilization occurred in 3% of daphnids when exposed to TiO_2_ of ∼200 nm, whereas a significantly (p = 0.0022) higher proportion (66%) were immobilized when treated with nTiO_2_ of ∼100 nm (Figure 1). After 96 h, the immobilization induced by ∼200 nm particles increased to 57%, while ∼100 nm nTiO_2_ particles caused a significantly (p = 0.0022) higher occurrence of immobilization (100%). No effects were observed in the control treatments.

### Time and dose dependency of nTiO_2_ toxicity and dissipation

**Figure 2:**
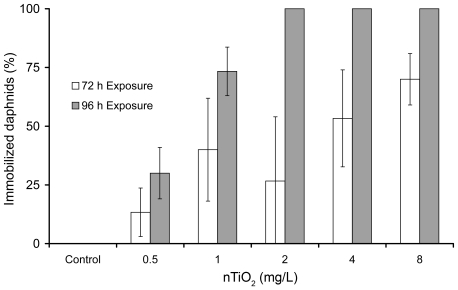
Mean immobilization (±SD; n = 6) of daphnids after 72 and 96 h of exposure to nominal concentrations of 0, 0.5, 1, 2, 4 and 8 mg nTiO_2_ (∼100 nm).

Similar to Exp. 1, no toxicity was observed at standard test durations. The results of Exp. 2 displayed dose- and time-dependent toxic effects (Figure 2). The calculated 72-h and 96-h EC_50_ values were 3.8 mg/L (95%; CI: 5.3–2.3) and 0.73 mg/L (95%; CI: 0.78–0.68), respectively.

**Figure 3:**
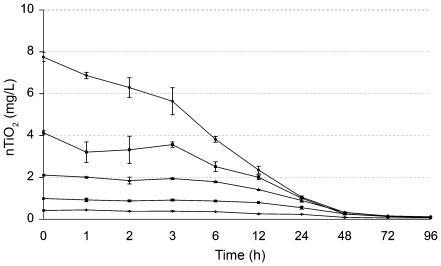
Dissipation of nTiO_2_ (∼100 nm) from the water phase. nTiO_2_ was measured as ^47^Ti, at initial concentrations of 0.5, 1, 2, 4, and 8 mg/L nTiO_2_ (ISO medium; means ± SD; n = 3).

Observations revealed a tendency for nTiO_2_ to form agglomerates and bind to organic and inorganic surfaces, resulting in dissipation from the water phase. All concentrations in Exp. 3 showed a fast decline of measurable nTiO_2_ concentrations from the water phase with higher initial concentrations dissipating faster than lower ones (Figure 3). None of the setups varying in initial concentration between 0.5 and 8 mg/L showed nTiO_2_ contents exceeding 1.06 mg/L after 24 h or 0.32 mg/L after 48 h. On the basis of these findings, we calculated time weighted average (TWA) values in the aqueous phase and recalculated the 96-h EC_50_ value of nTiO_2_ to be 0.24 mg/L (95%; CI: 0.22–0.26), based on measured aqueous-phase concentrations. Visual observations suggested that most of the dissipation of nTiO_2_ from the aqueous phase occurred via the formation of agglomerates due to the high ionic strength of the media. The agglomerates sink to the bottom of the test beakers and form a thin layer of TiO_2_.

### Mechanism of toxicity – biological surface coating

**Figure 4:**
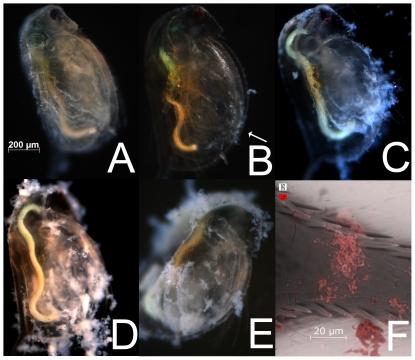
Development of biological surface coating in a 96-h toxicity test at 2 mg/L nTiO_2_ in ISO-medium. (A) 24 h, before 1^st^ molting; (B) 25 h, about 1 h after 1^st^ molting with arrow indicating renewed particle adhesions at filtration apparatus and the lower ventral gap; (C) 48 h; (D) 72 h; (E) 96 h; (F) ESEM-EDX picture of nTiO_2_ particle agglomerates colored in red on spine of *D. magna* following 48 h of exposure to, nTiO_2_ particles at 2 mg/L.

During all tests, NP showed a high tendency to bind to organic surfaces, resulting in a steadily growing layer of TiO_2_ on daphnids within the 96-h exposure period, resulting in a “biological surface coating” (Figure 4). The coating was visible within the first 24 h of exposure (Figure 4A) and completely disappeared with the first molting (shedding of shell). Biological surface coating reoccurred within 1 h after the first molting (4B) and continued steadily during the 96-h exposure period (Figure 4C to 4E). Because its enlarged surfaces and permanent movement caused high encounter rates with nTiO_2_, the filtering apparatus showed this particle adhesion phenomenon to a greater extent (arrow in 4B). SEM-EDX images confirmed that the particles involved in the biological surface coating were TiO_2_ (Figure 4F).

### Mechanism of toxicity – molting inhibition

**Figure 5:**
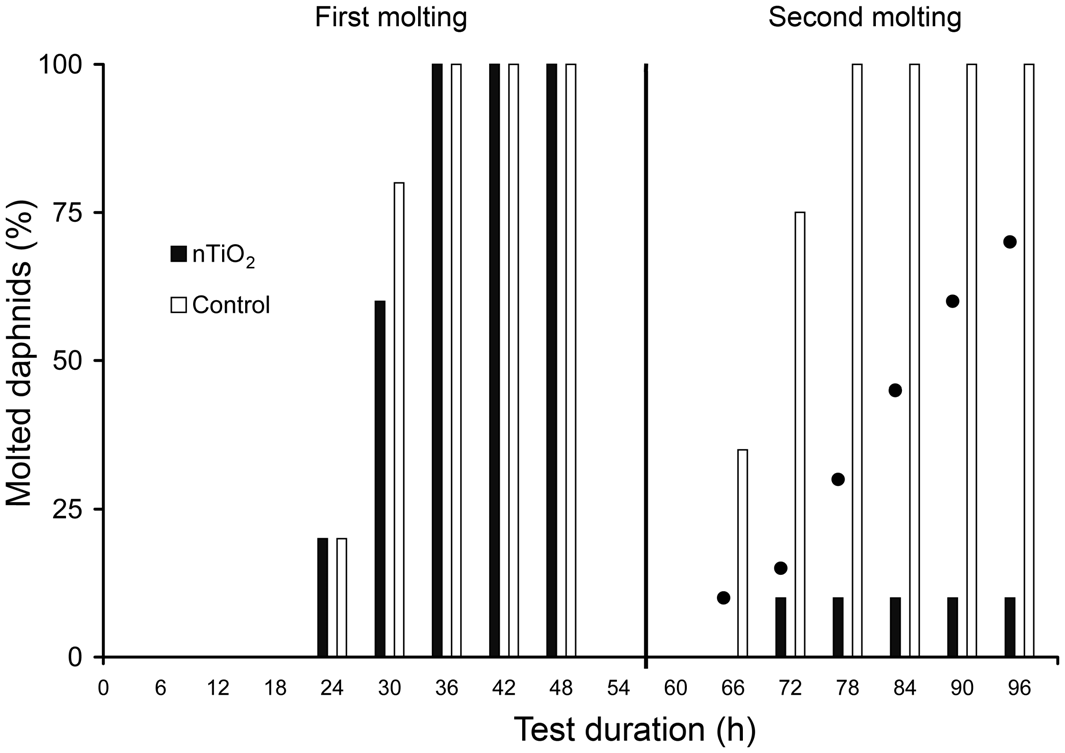
Molting success of juvenile *D. magna* (age ≤6 at 0 h) exposed to 2 mg/L nTiO_2_ (∼100 nm). Bars between 24 and 48 h on the left indicate the percentage of individuals passing the first molting, bars on the right, from 66 h onwards, represent those individuals passing the second molting. Black dots indicate the percentage of immobilized *D. magna* in the nTiO_2_ treatment.

During previous experiments, we found lower numbers of exuvia in test beakers containing nTiO_2_ and hypothesized an adverse effect of these NP on the molting success of *D. magna.* Hence, we conducted Exp. 4 with individually held specimens. The results revealed that all animals (age ≤6 h at test start) completed the first molting within 36 h without any substantial differences between animals exposed to 2 mg/L nTiO_2_ (∼100 nm) and the controls (Figure 5). The second molting occurred in 100% of control animals between 66 and 78 h after the start of the test, while animals exposed to nTiO_2_ showed a delay in molting and, most importantly, a significantly (p = 0.0065) lower molting success of only 10%. The TWA concentrations of TiO_2_ in the 2 mg/L setup at the time of the first (about 24 h) and second (about 72 h) molting were 1.5 and 0.8 mg/L, respectively (Figure 3).

## Discussion

### Size dependency of TiO_2_ toxicity

The size of the reactant surface is a key property of NP [7]. Thus, it was suggested that smaller NP are likely to cause higher toxicities at similar concentrations [22]. Nevertheless, almost no data from systematical investigations concerning particle size effects are available to date [14]. Comparing non-nanosized TiO_2_ (∼200 nm) with nTiO_2_ (∼100 nm), we showed a significantly elevated acute toxicity of nTiO_2_. nTiO_2_ was approximately twice as toxic within 96 h of exposure. To the best of our knowledge, this is the first study showing two different size classes of the same TiO_2_ stock suspension differing significantly in their toxicity. A recent study using TiO_2_ in a chronic exposure setup with *D. magna* implied that the observed effects on antioxidant enzyme activities increased with increasing particle sizes between 400 and 800 nm [23]. Drobne et al. [24] reported a higher toxicity of 25 nm versus 75 nm sized nTiO_2_ in terrestrial isopods. However, the actual size distributions were not characterized, and when the two size classes were compared, they were found to differ in crystalline structure (25 nm with 100% anatase and 75 nm with a mixture of anatase and rutile). In a recent study using the algae *Pseudokirchneriella subcapitata* Korshikov and nTiO_2_, no size-dependent differences in toxicity were detected for 10, 30, and 300 nm primary particles. These particles originated from different manufactures and had differing crystalline structures [25]. Pan et al. [26] found that gold NP of around 1 nm caused a higher toxicity to human and mouse cell lines than gold particles about 15 fold larger and attributed these findings to different uptake kinetics. For 100 nm particles, such as those used in the present study, the particle surface is about four times as high as for 200 nm particles. Therefore, the small particles may attach easier and bind more strongly to the daphnids exoskeleton, resulting in higher toxicity than the larger particles. Our findings indicate a different toxic response at equal concentrations based on mass, highlighting the importance of further research on the controversial question [22] regarding the importance of surface as a metric in NP toxicity testing. Another possible reason for a higher toxicity of nano sized particles is the higher rate of formation of reactive oxygen species as a result of the higher reactivity [2]. However, previous studies have not detected clear direct photocatalytic effects of nTiO_2_ on *D. magna* [15].

### Time and dose dependency of nTiO_2_ toxicity and dissipation

Our experiments revealed the importance of prolonging the test duration to 96 h, particularly in light of the absence of toxic effects after 48 h. For instance, in Exp. 2, we observed an immobilization rate of 100% at 2 mg/L after 96 h, while no toxicity was observed after 48 h in any concentration up to 8 mg/L. Based on the nominal concentrations, the 72- and 96-h EC_50_ values were 3.8 mg/L and 0.73 mg/L, respectively. This is far below the values reported in the majority of available studies on nTiO_2_ and *D. magna* [27]. Zhu et al. [9] found toxic effects of nTiO_2_ in a similar range (72-h EC_50_: 1.62 mg/L), although their findings are not exactly comparable with the results present in this study because they renewed their test media every 24 h and obtained a control mortality exceeding the validity criteria of the OECD standard test protocol 202 [10]. In contrast to all other studies focusing on nTiO_2_, Lovern and Klaper [8] found an EC_50_ value (48 h) of 5.5 mg/L but used a considerably smaller particle size (30 nm) than used in our study. However, no information was provided as to which primary particles were used in their study, and it remains unclear how the particle size distribution present in their toxicity tests, which was measured with transmission electron microscopy (TEM) using dried samples, varied or how strong the impact of sample preparation (airbrushing the suspension on the TEM grids) was on the TEM results.

The need for the adoption of standard tests for use with NP has often been voiced in a general way [16]. According to our results, the prolongation of the acute test duration to 96 h, as suggested for general chemical testing by Lazorchak et al. [18], would have the potential to considerably improve the assessment of acute NP toxicity.

For planktonic organisms, such as *D. magna,* it is likely that the exposure to NP takes place via the water phase. To the best of our knowledge, this is the first study reporting the temporal kinetics of NP concentrations in a test medium suspension, although the urgent requirement for better characterization of exposure during the actual test has been voiced repeatedly [28]. The observed decline of concentrations following second order kinetics is not surprising from a colloid scientific point of view, and other authors have also described nTiO_2_ agglomeration effects in culture media containing ions [29]. In seawater, Keller et al. [30] showed a higher agglomeration rate of nTiO_2_ at higher initial concentrations due to the increased probability of collisions between particles. From an ecotoxicological perspective, the fast aqueous phase dissipation implies that the actual concentrations to which the daphnids were exposed in the water phase over the 96-h test period were considerably lower than the nominal or measured initial concentrations. To consider this phenomenon, the usual procedure is to calculate TWA concentrations. However, this concept is used in chronic rather than acute toxicity testing [31]. Based on the 96-h TWA concentrations, the respective EC_50_ (96-h) is 0.24 mg/L, a factor of three lower than the value based on initial nominal concentrations.

This effect threshold results in a predicted no effect concentration of 0.24 µg/L, if calculated according to established procedures on risk assessment [32], including an assessment factor of 1000. Due to the fact that the maximum measured concentration of nTiO_2_ in urban runoff is about 0.073 µg/L [12] and a further dilution of urban runoff appears after entering surface water, it remains unlikely that nTiO_2_ levels will exceed the acute predicted no effect concentration. Although this leads to the assumption that current usage does not result in an acute risk, this might change based on the predicted increase in production and usage [1]. Moreover, further studies on the chronic toxicity of nTiO_2_ [9] may lead to lower effect thresholds.

### Mechanism of nTiO_2_ toxicity

As outlined previously, one of the most frequently used test organisms for nTiO_2_ in aquatic ecotoxicology is *D. magna*, and it seems surprising that there is still a huge gap in the knowledge concerning the potential mechanisms of nTiO_2_-mediated toxicity. Our results strongly suggest a mechanistic chain of events in NP-organism interactions, which has not been addressed thus far in the scientific literature. This chain of events involves biological surface coating in combination with a molting disruption in juvenile *D. magna*.

The coating of the daphnids' outer surfaces and filtering apparatus with TiO_2_ is a rapid process, occurring within the first 24 h after birth. During the first molting at about 24 h after birth, the daphnids shed their shell and the attached TiO_2_. However, only 1 h later, the progressing biological surface coating again leads to TiO_2_ agglomerates being attached to surface structures, such as the filtering apparatus (arrow in Figure 4B), a process which continues throughout the 96-h exposure period. The concept of the adhesion of NP to the surface of organisms is not entirely new [33] and has already been described for TiO_2_ and *D. magna* in a qualitative way [6]. However, concentrations of 40.5 mg/L TiO_2_, about 20 times higher than in the present study, have been used, and the observed surface adhesion was considerably lower. Adsorption of the material to the exterior surface of the organisms can be considered as the first step in the biological uptake of any NP. Although uptake of nTiO_2_ into *D. magna* has been observed [9] due to the low solubility and the low intrinsic toxic potential of titanium itself [34], it is likely that acute toxicity due to cellular, biochemical or macromolecular changes plays only a minor role. Furthermore, it has also been implied in the literature that the aggregate adhesion may cause physical effects and a loss of mobility [6]. From the results obtained in this study, it appears likely that such physical effects are of major importance. As displayed in Figure 4, substantial amounts of nTiO_2_ agglomerate within short time periods on the daphnid exoskeleton. Taking into account that the biological surface coating results in the increase of both the specific weight and the physical resistance during swimming movements, it is likely that the energy demand strongly increases. However, additional experiments revealed slightly elevated levels of lipid content (9.3% average increase compared to control; n = 7; measured using sulfo-phospho-vanillin reaction) and total energy (52.6% average increase compared to control; n = 9; measured using differential scanning calorimetry) in juvenile *D. magna* after 48 h of exposure to 2 mg/L nTiO_2_. It should be further noted that due to the increased probability of collisions between particles [30], the extent of biological surface coating should be even greater for smaller sized NP.

Apart from zooplankton, NP adhesion to biological surfaces has also been shown in other organisms. For instance, similar phenomena have been found for carbon nanotubes and the gill mucus of rainbow trout [35] and have also been implied for TiO_2_ and rainbow trout gill surfaces [36]. Potential sorption has also been reported for phytoplankton by Aruoja et al. [37], who observed an entrapping of algae cells (*P. subcapitata*) by TiO_2_ aggregates formed during the 72-h toxicity tests. Being a zooplankton filter feeder, *D. magna* relies on phytoplankton as a food source, which might lead to additional indirect negative effects. However, these effects have not been studied so far [6]. Apart from the adhesion to the exoskeleton, sublethal endpoints for ingestion and filtration rates of *D. magna* have been documented in specimens exposed to nTiO_2_ for 5 h at TiO_2_ concentrations of 0.5 mg/L [38]. In addition, recent publications revealed the potential of NP to be transported within the food web [39], [40], leading to a dietary exposure of aquatic organisms on higher trophic levels.

Our results clearly show that a 96-h exposure to 2 mg/L of ∼100 nm nTiO_2_ particles significantly reduces the success of the second molting to 10%, compared to a 100% molting success in the control. This molting disruption, ultimately leading to the observed high levels of immobility and mortality, was only visible during the extended exposure period of 96 h, while no difference was detected between the TiO_2_ and control setup during the first molting (within the initial 48 h of the test). This could explain why, as discussed above, so many studies did not observe any substantial toxicity of nTiO_2_ to *D. magna* during the standard 48-h test duration. Apart from the initial concentration, the TWA concentration at the time of the first (1.5 mg/L) and second molting (0.8 mg/L) might have been important for the observed effects. During the second molting, the daphnids might have been particularly susceptible to nTiO_2_ toxicity. A strong negative effect of NP on the molting of *D. magna* has not been reported previously in the scientific literature. When exposing *D. magna* to different fullerene concentrations over 21 days, a slight, yet significant, molting delay was found at 2.5 mg/L [41]. However, no effect was observed during the same study at lower concentrations of 0.5 and 1 mg/L or at a higher concentration of 5 mg/L. Because the molting of *Daphnia* is intimately associated with growth and reproduction, a delay or disruption of molting translates directly into reduced reproduction rates [42].

The fact that the second molting of juvenile daphnids is disrupted, means that both the standard acute and the standard chronic *D. magna* tests are not suitable for the detection of the aquatic toxicity of nTiO_2_. In the acute test, the test duration does not cover the second molting. In the chronic test, juveniles are regularly removed from the test system before they perform their second molting and only the absolute number of offspring is considered in the evaluation.

In conclusion, our study shows that slightly elongated test durations reveal toxic effects at low concentrations of nTiO_2_ and thus presents a reasonable modification of standard test systems for NP. In addition, we revealed a negative impact of nTiO_2_ on the molting and development of *D. magna*, which is likely to be connected to biological surface coating as a general pathway of physical NP toxicity. Because this pathway might be ubiquitous for aquatic organisms, the resulting adverse effects on food webs and ecosystems may be widespread.
